# Evaluation of psychometric properties of dietary habits, lifestyle, food frequency consumption, and nutritional beliefs (KomPAN) questionnaire in Iranian adults

**DOI:** 10.3389/fpubh.2022.1049909

**Published:** 2022-11-25

**Authors:** Kiyana Saadati, Fakhreddin Chaboksavar, Khadije Jahangasht Ghoozlu, Abbas Shamsalinia, Mohammad Reza Kordbageri, Reza Ghadimi, Zeinab Porasgari, Fatemeh Ghaffari

**Affiliations:** ^1^Medicine Department, Mazandaran University of Medical Sciences, Sari, Iran; ^2^Nursing and Midwifery Department, Nursing Care Research Center, Health Research Institute, Babol University of Medical Sciences, Babol, Iran; ^3^Statistic Department, Shahid Beheshti University (SBU), Tehran, Iran; ^4^Social Determinants of Health Research Center, Health Research Institute, Babol University of Medical Sciences, Babol, Iran; ^5^Department of Sport Nutrition, Islamic Azad University, Central Tehran Branch, Tehran, Iran

**Keywords:** diet quality index, dietary habits, lifestyle, nutrition knowledge, psychometric evaluation

## Abstract

**Background:**

Adherence to unhealthy dietary patterns is a major cause of overweight and obesity in adults. Therefore, it is recommended that assessment and modification of unhealthy lifestyle should be included in prevention programs. To achieve this goal, it is necessary to evaluate the status of dietary patterns in adults with valid and reliable tools. Thus, the aims of the present study were to translate the KomPAN questionnaire, evaluate its psychometric properties in Iranian adults and measure 4 dietary indices including high-saturated-fats-Diet-Index-8 (hSFDI-8), high-Sugar- Diet-Index-4 (hSDI-4), low-Glycaemic-Diet-Index-4 (LGIDI-4) and high- Glycaemic-Diet-Index-7 (hGIDI-7) based on 3 groups of body mass index (BMI) (BMI = 18.5–24.9, BMI = 25–29.9 and BMI ≥ 30), gender, educational level, income status, and age.

**Methods:**

The KomPAN questionnaire included 4 scales nutrition beliefs (NB), lifestyle, food frequency consumption (FFC), dietary habits (DH) and after its translation from English into Persian, the psychometric properties of all 4 scales (face and content validity) were evaluated. For both FFC and NB scales, the construct validity was assessed through exploratory factor analysis (EFA), confirmatory factor analysis (CFA) and convergent and discriminant validity, the internal consistency was evaluated using the Cronbach's alpha coefficient, McDonald's omega (Ω) and Theta coefficient (θ), as well as the stability was assessed *via* intraclass correlation coefficient (ICC). Cross-classification and Kappa statistics were evaluated for both DH and lifestyle scales. Then, 4 dietary indices were measured in terms of demographic variables.

**Results:**

The cross-classification of DH (93.96%) and lifestyle (95.87%) scales indicated the percentage of correct classification in the test-retest scales. The Kappa statistic was >0.4 and its value was acceptable. The mean Kappa statistics were 0.734 and 0.865 for the DH and lifestyle scales, respectively. The fit indices showed that the two-factor construct of the FFC scale and the one-factor construct of the NB scale had a good and acceptable fit among the Iranian adults. The FFC and NB scales had acceptable internal consistency and stability.

**Conclusion:**

It is recommended that other researchers use the KomPAN questionnaire to identify DH, FFC, NB and lifestyle as well as measure diet quality scores in the adult community.

## Background

A varied, balanced and healthy dietary pattern is one of the most important aspects of a lifestyle that is useful for maintaining health and preventing chronic diseases (1). A rational diet is a key determinant of adult health. However, the prevalence of unhealthy food choices is high among adults (2). The results of studies show that unhealthy dietary patterns are associated with obesity, insulin resistance, metabolic syndrome and other risk factors for cardiovascular disease (3–5).

In recent years, the use of dietary pattern analysis in various groups including adults has received much attention. Dietary patterns can summarize the combined effects of common foods used by the community (6) and reflect that the food interests and preferences of individuals are influenced by cultural, economic, social and lifestyle factors (7, 8).

The American Dietetic Association in healthful eating messages to the public suggests that it should be emphasized dietary patterns rather than foods or meals because assessing dietary patterns can help assess nutrients or the amount of food received (9). On the other hand, since nutrients and foods are not consumed separately, nutritionists have also suggested that in order to achieve a broader picture of the diet, it is necessary to evaluate a person's dietary patterns (10).

Studies have demonstrated that adherence to unhealthy dietary patterns such as high-calorie foods, increased consumption of fast foods and sedentary lifestyle is a major cause of overweight and obesity in adults (5, 7).Therefore, it is recommended that assessment and modification of unhealthy lifestyle should be included in prevention programs including increasing physical activity, increasing the quality of the diet, modifying the diet such as consumption of foods with the low glycemic index, saturated fat, low sugar and weight loss (11). To do so, it is necessary to evaluate the status of dietary patterns in different communities with valid and reliable tools. One of the available tools is the Dietary Habits, Lifestyle, food frequency consumption and Nutritional Beliefs Questionnaire (KomPAN questionnaire), which was developed in 2014 by the Committee of Human Nutrition, Polish Academy of Science, in two interviewer-administered (IA-Q) and self-administered (SA-Q) versions (12). The KomPAN questionnaire is a reliable and valid tool and has been used in some sections of several published studies (11–14). One of the advantages of using this tool is that the diet quality scores (DQS) can be calculated by evaluating the food frequency consumption (FFC). DQS is a very important predictor of non-communicable diseases (15), and its analysis reflects people's dietary patterns in real life and their dietary habits (11). Calculating DQS may be particularly useful in nutrition education interventions or clinical settings (16, 17). Other benefits of using this tool are simplicity, applicability, nutritional habits and beliefs, comprehensiveness in assessing the various dimensions of dietary patterns, as well as lifestyle in adults. The KomPAN questionnaire is the first questionnaire to evaluate dietary habits (DH), FFC, nutrition beliefs (NB) and lifestyle that has been confirmed to be reproducible in people of a wide age range (in healthy individuals and individuals with chronic diseases) (12). Although the findings of the research lead to valid and reliable evidence if the variables are measured using tools consistent with the culture of communities (18). The KomPAN questionnaire is not localized in Iranian adults. The psychometric properties of this tool have not been evaluated in other studies.

## Objectives

Thus, the aims of the present study were to translate the Dietary Habits, Lifestyle, food frequency consumption and Nutritional Beliefs Questionnaire (KomPAN questionnaire), evaluate its psychometric properties in Iranian adults and measure 4 dietary indices including high-saturated-fats-Diet-Index-8 (hSFDI-8), high-Sugar-Diet-Index-4 (hSDI-4), low-Glycaemic-Diet-Index-4 (LGIDI-4) and high- Glycaemic-Diet-Index-7 (hGIDI-7) based on 3 groups of body mass index (BMI) (BMI = 18.5–24.9, BMI = 25–29.9 and BMI ≥ 30), gender, educational level, income status, and age.

## Materials and methods

### Design and setting

In this validation study, the setting of the study was comprehensive health service centers in the cities of Mazandaran province, Iran. The choice of this setting was due to the availability of samples with relatively common cultural structures and lifestyles. This study with the ethics code of IR.MUBABOL.HRI.REC.1400.046 was conducted in 2022.

### Measures

In the current study, two questionnaires were used to collect data:

**Demographic characteristics questionnaire**: This questionnaire included age, gender, educational level, marital status, place of residence, occupational status, economic status, number of people in the household, weight, height and BMI.
**KomPAN questionnaire:** In the present study, the self-administered questionnaire (SA-Q) version of this questionnaire was used. In the ongoing study, according to the nutritional culture, lifestyle of the target community and opinions of the panel of experts (research team), items were added to different parts of the KomPAN questionnaire or items were combined with each other, which was explained in each section. KomPAN questionnaire consisted of 4 scales.

#### Dietary habits (DH)

DH contained 10 multiple-choice items (one or more than one correct answer) and investigated regular consumption of meals, snacks, soft drinks or ready meals, sweets and so on.After translating the tool and checking its items by the research team, 4 items were added to the DH scale according to the food culture of Iranian adult society.

In the current study, four food groups including “Do you use smoked foods such as smoked rice and smoked fish?,” “Do you use food seasonings such as sour, salt, pepper, cinnamon and ginger?,” “Do you add rice bran to your food? ” and “What kind of snack do you usually eat between meals during the day?” were added to this scale. The data of this scale had qualitative characteristics and its analysis was performed by cross-classification analysis and Kappa statistics.

#### Food frequency consumption (FFC)

FFC consisted of 33 items including cereals (4 items), fruits/vegetables/legumes/potatoes (5 items), dairy products (4 items), meat/fish/eggs (6 items), fats (3 items), drinks (7 items), sweets and other products (4 items). The FFC was determined by respondents based on a 6-point Likert scale (never, 1–3 times a month, once a week, several times a week, once a day or several times a day). Numerical values were assigned to FFC (once a day = 1, few times a day = 2, few times a week = 0.5, once a week = 0.14, 1–3, times a month = 0.06, and never = 0).

In the present study, the item “How often do you eat fish?” was combined with the item “How often do you eat white meat example chicken, turkey and rabbit?” as well as the item “How often do you consume nuts, sunflower seeds, pistachios, hazelnuts and walnuts?” was added to this scale. Kowalkowska et al. (12), according to FFC items, determined two nutritional indices including pro-Healthy-Diet-Index (pHDI) (whole-wheat bread; whole-wheat cereals, oatmeal or whole-wheat pasta; milk; fermented milk drinks; cottage cheese; white meat; fish; dishes with legumes; fruits and vegetables) and non-Healthy-Diet-Index (nHDI) (white bread; white rice, pasta or fine-ground groats; fast food; fried dishes; butter; lard; cheese; cold meats, smoked sausages or hot-dogs; red meat dishes; sweets; tinned meats; sweetened carbonated and non-carbonated drinks; energy drinks and alcoholic beverages). In the current study, *via* reviewing the texts and using the opinions of nutritionists, the items of instant soups or ready-made soups (example tinned, jar and concentrates), tinned (jar) vegetables (example pickles), still beverages and sweetened hot beverages were added to nHDI as well as the items of vegetable oils, eggs, vegetable juices, fruit and vegetable juices, potato and water were added to pHDI. Therefore, the number of items and score levels were changed as follows:

**pHDI-15:** The pHDI-15 included whole-wheat bread; whole-wheat cereals, oatmeal or whole wheat pasta; vegetable oils; milk; fermented milk drinks; cottage cheese; white meat; nuts; vegetable juices, fruit and vegetable juices; eggs; dishes with legumes; potato; fruits; vegetables; vegetable juices or fruit juices and water. The total score range was 0–30 points divided into three categories: low (0–10.0), moderate (10.1–20.0) and high (20.1–30.0). PHDI is interpreted in such a way that the higher the value represents the greater the intensity of the FFC desirable characteristics.

**nHDI-18**: It consisted of white bread; white rice, pasta or fine-ground groats; fast food; fried dishes; butter; lard; cheese; cold meats, smoked sausages or hot dogs; red meat dishes; sweets; instant soups or ready-made soups (example tinned, jar and concentrates); tinned meats; tinned (jar) vegetables (example pickles), still beverages; sweetened hot beverages sweetened carbonated and non-carbonated drinks; energy drinks and alcoholic beverages. The total score range was 0–36 points categorized into three categories: low (0–12.0), moderate (12.1–24.0) and high (24.1–36.0).

The interpretation of nHDI is such that the higher the value represents the greater the intensity of the FFC undesirable characteristics. In the present study, according to the classification of Bykowska-Derda et al. (19) four indices including hGIDI-7, LGIDI-4, hSDI-4 and hSFDI-8 were evaluated (11). Moreover, the values of these indices were compared based on 3 groups of normal (BMI = 18.5–24.9), overweight (BMI = 25–29.9) and obese (BMI ≥ 30) BMIs, gender, educational level, income status, and age:

**hGIDI-7:** The hGIDI**-7** was composed of items including white bread; white rice, pasta or fine-ground groats; fruits, sweets, juices, sweetened hot drinks as well as sweetened carbonated and non-carbonated drinks.
**LGIDI-4:** The LGIDI-4 comprised items of whole-wheat bread; whole-wheat cereals, oatmeal or whole-wheat pasta; dishes with legumes and vegetables.
**hSDI-4:** The hSDI-4 involved items such as sweets, juices, sweetened hot drinks as well as sweetened carbonated and non-carbonated drinks.
**hSFDI-8:** The hSFDI-8 included items of fast food; fried dishes; butter; lard; cheese; cold meats, smoked sausages or hot dogs; red meat dishes and tinned meat. Based on the following formula and using the data of this scale, the DQS was calculated as dietary indexes (20).

$$\begin{array}{l} \text{Diet Quality Index}\;=\;\frac{100^{*}\sum^{\text{​}}\text{A}}{\sum^{\text{​}}\text{B}}\;[\%] \end{array}$$

Where A is the sum of the reported daily intake of all items listed in specific food groups (e.g., low GI), for example, Σ = 0 + 0·14 + 0·06 + 0·5. B is the sum of the maximum possible to report daily intake of the same (low GI) foods, determined for one product as 2 (e.g., Σ = 2 + 2 + 2 + 2). The total score of diet quality was from 0 to 100 (20). The total score of nutritional trait intensity was classified into low (0–33.32% points), moderate (33.33–66.65% points) and high (66.66–100% points) (11).

#### Nutrition beliefs (NB)

NB consisted of 25 items, each item is scored with 3 response categories (true = 1, false = 0 and not sure = 0). The scores of all items were summed (the total NB score range: 0–25 points). The response to the items was divided into three categories: insufficient (0–8), sufficient (9–16) and good (17–25) (21).

#### Lifestyle

This scale comprised 16 items that included different aspects of lifestyle such as diet, drinking alcohol, smoking, sleep, screen time, recreational physical activity and type of water consumed. Lifestyle items were scored differently.

For example, the physical activity was scored based on a 3-point Likert scale including low = 1, moderate = 2 and high = 3, or self-declared by the respondent of nutritional knowledge was scored based on a 4-point Likert scale including insufficient, sufficient, good and very good (12). In the current study, two items including “Are you currently using drugs such as poppy, opium, opium juice (Shireh), methadone, bupropion and heroin?” and “Are you currently using industrial addictive substances such as hashish, marijuana, drug flowers, crack and methamphetamine” were added to the lifestyle. NB scale data had qualitative characteristics and were analyzed by cross-classification analysis and Kappa statistics.

### Cross-cultural adaptation of KomPAN questionnaire and content validity

In the development of the Persian version of the KomPAN questionnaire based on the WHO protocol (2015), the forward-backward translation technique was used (12). Firstly, written permission was obtained from Professor Marzena Jezewska-Zychowicz who designed this tool *via* email for translating and validating the tool. The cross-cultural adaptation process of the KomPAN questionnaire and the evaluation of content validity were done in three steps:

**Step 1: Forward translation (Translation of the original questionnaire):**
The English KomPAN questionnaire was translated into Persian by two translators, independently.
**Step 2: Reconciliation of forward translations (Synthesis of the translations):**
The two Persian translations were reviewed by the research team, and finally, a single Persian version of the questionnaire was prepared,
**Step 3 Backward translation (Back translation of the consolidated version):**
The backward translation (from Persian into English) was performed independently by two other translators. The two English (backward) translations were reviewed and compared with the original (English) questionnaire by the research team. After the necessary corrections, the final version of the questionnaire was sent to Marzena Jezewska-Zychowicz *via* email for approval.

### Pre-test

The forward translation (final version) of the KomPAN questionnaire was completed by 30 adults selected based on inclusion criteria. This part of this study was done by the corresponding author. The reactions of these individuals during responding to items such as long pauses in responding to each item, changes in response to items and symptoms such as confusion when responding to items were examined. All adults completed the questionnaire in the pre-test stage. Some of the items were not easy for them to understand; therefore, some modifications were made to items based on their suggestions.

### Assessment of the validity (face and content construct), convergent and divergent validity, reliability, cross-classification, and Kappa of the KomPAN questionnaire

#### Face validity

Face validity was qualitatively and quantitatively evaluated for all 4 scales of the KomPAN questionnaire. For qualitative face validity, 10 persons of the target group were asked to comment on the levels of difficulty, appropriateness and ambiguity of each item through individual and face-to-face interviews. Proposed corrections were made to the items. In the current study, the impact score was calculated using the following formula:


$$\begin{array}{l} \text{Impact item}=\text{frequency }\left(\%\right)\times \text{ importance} \end{array}$$

Items with ≥ 1.5 were appropriate, and other items were removed (22).

#### Content validity

Content validity was assessed both qualitatively and quantitatively for all 4 scales of the KomPAN questionnaire. To do so, the questionnaire was sent to 10 experts (8 persons who had experience in performing qualitative studies and making tools as well as 3 nutritionists) *via* email. These individuals were asked to evaluate grammar, wording, item allocation and scaling of tools. All changes suggested by experts were made to the items. Content validity ratio (CVR) and content validity index (CVI) were evaluated in the quantitative part (23). To calculate the CVR, experts (the same persons invited to review the quality of the content validity) were asked to comment on each item based on a three-point scale (from “not essential” to “essential”). Then, the CVR was calculated using the following formula:


$$\begin{array}{l} \text{CVR }=\;\frac{\text{ne}-\left(\frac{\text{N}}{2}\right)}{\left(\frac{\text{N}}{2}\right)} \end{array}$$

ne = the number of experts selected an item “essential,” N = the total number of experts evaluated all items.

The minimum acceptable CVR was determined based on the Lawshe (1975) table. The number of experts was 10, so the acceptable value of CVR was ≥ 0.62 (24).

To evaluate CVI, Waltz & Bausell method was used (25). Thus, 10 experts (the same ones invited to assess the quality of the content validity) were asked to determine the relevance of each item based on a four-point Likert scale ranging from 1 = irrelevant, 2 = somewhat relevant, 3 = quite relevant to 4 = highly relevant. The CVI was then calculated using the following formula:


$$\begin{array}{l} \text{CVI }=\;\frac{\text{The number of the experts who checked option }3\text{ and }4\;}{\text{The total number of experts}} \end{array}$$

The acceptable value of CVI was >0.79, and if the CVI value was between 0.70 and 0.79, the item was revised (25).

#### Construct validity

For both NB and FFC scales, the construct validity was assessed using Exploratory Factor Analysis (EFA) and Confirmatory Factor Analysis (CFA).

A cross-sectional study was conducted to evaluate the construct validity. The study population included all adults referred to comprehensive health service centers. Samples were selected using the convenient sampling method. Inclusion criteria were 18–60-year-old persons having literacy, no chronic diseases (having no specific diet), no allergy to one type of food, no specific diet program (People working in a system that uses a special diet.), no anorexia nervosa and no bulimia nervosa. Exclusion criteria included not completing the questionnaires completely and the person who refused to continue working with the research team. To evaluate the validation of two scales of FFC and NB, all samples (*N* = 1,400) were randomly divided into two subgroups of 700 persons. The first subgroup included 386 females and 314 males (M_age_ = 37.92, SD = 11.59; M_BMI_ = 26.60, SD = 6.01) and the second subgroup was 335 women and 365 men (M_age_ = 37.57, SD = 10.49; M_BMI_ = 27.53, SD = 4.75). Only for both NB and FFC scales, the construct validity was evaluated using EFA and CFA:

### EFA

EFA was performed on the samples of the first subgroup (*N* = 700) for FFC and NB instruments. The Kaiser-Meyer-Olkin (KMO) and Bartlett's sphericity tests were utilized to assess sample adequacy and sphericity, respectively. Then, the latent factors of both sections were extracted using the principal axis factoring (PAF) method, Varimax rotation and scree plot. The presence of a single item in the factor was ~0.3 based on the following formula:


$$\begin{array}{l} \text{CV }=\;5.152\;÷\;\sqrt{\left(\text{n }-\;2\right)} \end{array}$$

The CV is the number of extractable factors and n is the sample size of the study.

Therefore, items with factor loadings lower than 0.3 are eliminated in the EFA (26).

### CFA

CFA was conducted on the samples of the second subgroup (N = 700). Model fitting was carried out using the goodness of fit indices (GFI), Satorra–Bentler scaled chi-square test (S–B χ^2^), comparative fit index (CFI), Tucker–Lewis index (TLI), standardized root mean square residual (SRMR), root mean square error of approximation (RMSEA) and confidence interval (CI) of 90%. The cut-off points of CFI and TLI>0.9, SRMR ≤ 0.08 and RMSEA ≤ 0.08 were considered as the acceptable limit. The cut-off points of CFI and TLI>0.9 as well as SRMR and RMSEA ≤ 0.08 were considered acceptable.

#### Convergent and discriminant validity

At this stage, the correlation between FFC and NB with age, gender and BMI status was investigated. Positive and significant values (>0.3) indicated appropriate convergent validity (27). In addition, two indices of average variance extracted (AVE) and construct reliability (CR) were used to evaluate the convergent validity. Values of AVE > 0.5 and CR > AVE represented acceptable convergent validity (28).

#### Reliability assessment

In the present study, several methods were utilized to evaluate the reliability:

Cross-classification analysis and Kappa statistics were applied to assess the reliability of DH and lifestyle scales. Kappa values >0.4 were considered acceptable agreement (29). To measure cross-classification and kappa statistics, 150 participants (73 females and 77 males (M_age_ = 37.75, SD = 10.01; M_BMI_ = 26.98, SD = 3.31) were selected based on inclusion criteria, and on two occasions (with an interval of 4 weeks), they responded to items on the dietary habits and lifestyle scales.

The reliability of the FFC and NB scales was evaluated using the internal consistency [Cronbach's alpha coefficient (α), McDonald's omega (Ω) and Theta coefficient (θ)] and stability reliability [intraclass correlation coefficient (ICC)]. ICC >0.8 (30) and α > 0.7 (31) and were considered acceptable levels.

### Data analysis

In the present study, to evaluate EFA, the R_4.5_ software (Psych and Polycor packages) was used for validation of two FCC and NB scales, and to assess CFA, the MPlus6.1 software was applied. Convergent and discriminant validity was performed through the Pearson correlation test and the Fornell-Larcker criterion (1981) (28). The cross-classification analysis and Kappa statistics in SPSS26 were used to validate DH and lifestyle scales.

## Results

### Characteristics of the participants

The results suggested that the mean age, weight, height and BMI of participants were 37.75 ± 11.05 years, 76.37 ± 13.37 kg, 168.30 ± 9.75 cm and 26.60 ± 6.01, respectively. Among these adults, 51.5% and 48.5% were females and males. Moreover, 56.4, 28.3, 7.8 and 7.5% were married, single, widowed and divorced, respectively. Among them, 48.4 and 51.6% were employed and unemployed as well as 42.5 and 57.5% had diploma and academic degrees, respectively. The economic status of 73.6 and 26.4% was sufficient and insufficient, respectively. Totally, 80 and 20% of them lived in urban and rural areas. In addition, 37.8% of individuals had normal BMI, as well as 37.6 and 24.6% were overweight and obese, respectively. None of them had BMI < 18.5 (underweight).

#### Dietary habits

The DH scale had 14 items, of which 6 items were analyzed and the other items were not analyzed because each participant could choose more than one option. According to test-retest results, the percentage of participants classified at the item level was on average 93.96%. So that among the studied adults, the highest cross-classification agreement was 98 and 95.3%, for the items “What type of milk (pasteurized high- or low-fat milk or whole milk) and dairy products do you usually consume?” and “Do you add sweeteners to hot drinks like tea, hot chocolate and coffee?,” respectively. Cross-classification analysis showed that on the DH scale, the percentage of correct classification was high, and the percentage of misclassification was very low among the studied persons. Kappa value ranged from 0.968 (for item “What type of milk (pasteurized high- or low-fat milk or whole milk) and dairy products do you usually consume?”) to 0.851 (for item “Do you use food seasonings such as sour, salt, pepper, cinnamon and ginger?”), respectively. The Kappa statistic was >0.4 for all analyzed items and its value was acceptable (Table 1).

**Table 1:** Agreement and misclassification in test-retest for dietary habits scale.

**No**.	**Questionnaire items**	**Cat**.	**Total agreement**	**Misclassification**			**Kappa**
				**1 ±cat**.	**2 ±cat**.	**3 ±cat**.	
1	What type of milk (pasteurized high- or low-fat milk or whole milk) and dairy products do you usually consume?	3	98	2			0.968
5	Do you add sweeteners to hot drinks like tea, hot chocolate and coffee?	4	95.3	0.67	2	2	0.927
6	Do you use smoked foods such as smoked rice and smoked fish?	3	93.3	4	2	0.7	0.894
7	Do you use food seasonings such as sour, salt, pepper, cinnamon and ginger?	2	93.3	6.7			0.851
8	Do you add salt to meals after cooking?	3	92.6		4	3.4	0.879
9	Do you add rice bran to your food?	3	91.3	7.3	0.7	0.7	0.856

No., item number in the questionnaire. Cat., number of response categories in the question. Evaluated in 3 categories: low (0–33 points), moderate (34–66 points), high (67–100 points).

### Food frequency consumption

#### Construct validity

##### EFA

The results of evaluating sampling adequacy indices (KMO) (0.892) and Bartlett's sphericity test (χ^2^ = 13,480.37, *P* < 0.001) indicated that the data were suitable for EFA. Two factors (nHDI and pHDI) with eigenvalue (λ) values >1 were identified for the FFC scale and confirmed based on the scree plot diagram. The data were rotated by Varimax rotation, and in total, two factors of FFC were 44.85% of the total variance. Totally, the first 15-item factor “pHDI” and the second 16-item factor “nHDI” allocated 25.72% (λ = 7.973) and 19.133% (λ = 5.931) of the total variance, respectively. Additionally, there was a weak correlation (< 0.3) between the two factors. The findings revealed that factor loadings of all items (except for two items “How often do you use lard to flavor your bread or for cooking? (Q8)” and “How often do you eat tinned meats? (Q25)”) were >0.3; therefore, these two items were deleted. The correlation between all items and the total score was >0.3 (Table 2).

**Table 2:** Exploratory and confirmatory factor analyses of the food frequency consumption scale.

**No**	**Items**	**Internal consistency** **1043(α; ITC)**	**EFA Sample 1** ***n*** = **700**			**CFA Sample 2** ***n*** = **700**	
			**Factor loading**	**h^2^**	**Eigenvalue (%variance explained)**	**λ_X_**	**(AVE; CR)**
	**pHDI**						
2	How often do you consume whole-wheat breads such as homemade bread, barbari, sangak, taftoon and toast?	(0.936; 0.666)	0.692	0.497	7.973 (25.721%)	0.68	0.525;0.942
4	How often do you consume whole cereals such as oats, barley and wheat (whole or oatmeal), corn, popcorn and brown rice?	(0.935; 0.703)	0.724	0.528		0.72	
9	How often do you consume vegetable oils (such as sesame oil, olive oil, sunflower oil, canola oil), animal butter and margarine butter for cooking?	(0.936; 0.669)	0.693	0.482		0.70	
10	How often do you drink regular milk (plain milk without additives) or flavored milks such as cocoa milk, coffee milk, date milk, banana milk, honey milk and strawberry milk?	(0.933; 0.801)	0.828	0.686		0.84	
11	How often do you consume dairy products such as yogurt, buttermilk, curd and ice cream?	(0.934; 0.757)	0.779	0.607		0.77	
12	How often do you use high-fat cheese?	(0.936; 0.669)	0.697	0.508		0.70	
16	How often do you eat white meat such as chicken, ostrich, turkey, quail and partridge and seafood such as fish and shrimp?	(0.932; 0.820)	0.846	0.718		0.84	
17	How often do you consume nuts, sunflower seeds, pistachios, hazelnuts and walnuts?	(0.934; 0.726)	0.742	0.552		0.74	
18	How often do you eat bird eggs such as chicken, quail, partridge, duck and goose eggs?	(0.934; 0.753)	0.775	0.604		0.77	
19	How often do you eat legumes such as split pea, bean, chickpea, mung bean, lentil, red lentil, broad bean and soybean?	(0.941; 0.523)	0.537	0.291		0.54	
20	How often do you eat potatoes but not crisps?	(0.933; 0.798)	0.825	0.681		0.81	
21	How often do you eat fruit (raw and dried fruit)?	(0.936; 0.674)	0.706	0.498		0.69	
22	How often do you eat raw vegetables (lettuce, carrots, cabbage, pumpkin, onions, mushrooms, cauliflower, broccoli, celery, spinach, vegetables, tomatoes, cucumbers) and cooked vegetables?	(0.934; 0.734)	0.765	0.585		0.75	
28	How often do you drink vegetable juices or fruit and vegetable juices?	(0.937; 0.622)	0.642	0.417		0.65	
32	How often do you drink water?	(0.939; 0.577)	0.601	0.361		0.61	
	**nHDI**				5.931 (19.133%)		0.535;0.948
1	How often do you use white bread (lavash) or bakery products such as baguettes and toast?	(0.889; 0.556)	0.601	0.363		0.65	
3	How often do you use white rice or white pasta?	(0.891; 0.479)	0.497	0.247		0.70	
5	How often do you eat fast food such as French fries, burgers and pizza?	(0.881; 0.768)	0.820	0.672		0.81	
6	How often do you consume fried foods such as fried meat or fried sweets such as dumplings?	(0.887; 0.580)	0.606	0.368		0.77	
7	How often do you use butter (animal or vegetable) or oils such as homemade oils to flavor your bread?	(0.893; 0.422)	0.444	0.198		0.62	
13	How often do you use processed cheeses?	(0.885; 0.652)	0.702	0.492		0.75	
14	How often do you consume smoked sausages, cold meats and hot dogs?	(0.890; 0.517)	0.542	0.298		0.70	
15	How often do you consume red meat and offal of livestock and poultry such as heart, liver, gizzard, Kalle pache (Khash), kidney, tripe and abomasum?	(0.883; 0.725)	0.783	0.615		0.81	
23	How often do you consume sweets such as pastries, biscuits, Sohan, cakes, chocolates, jams and fruit compotes?	(0.883; 0.690)	0.745	0.556		0.78	
24	How often do you use ready-made or instant soups such as noodle, mushroom, chicken or vegetable soups?	(0.890; 0.514)	0.522	0.276		0.74	
26	How often do you eat tinned (jar) vegetables such as pickles and peas?	(0.889; 0.522)	0.531	0.282		0.65	
27	How often do you drink still beverages	(0.883; 0.692)	0.741	0.551		0.77	
29	How often do you drink sweetened hot beverages such as black tea, coffee and herbal or fruit teas?	(0.889; 0.538)	0.577	0.335		0.62	
30	How often do you drink sweetened carbonated and non-carbonated drinks like Coca, Pepsi, Lemonade or Fanta?	(0.891; 0.484)	0.500	0.250		0.75	
31	How often do you drink energy drinks like Red Bull, Life, Monster or Rockstar?	(0.896; 0.288)	0.536	0.089		0.84	
33	How often do you drink alcoholic beverages?	(0.890; 0.500)	0.545	0.298		0.70	

EFA, Exploratory Factor Analysis; CFA, Confirmatory Factor Analysis; Internal Consistency (Cronbach's Alpha if item deleted and Item-Total Correlation [ITC]); λx, standardized coefficients; h2, Communalities.

##### CFA

**Figure 1:**
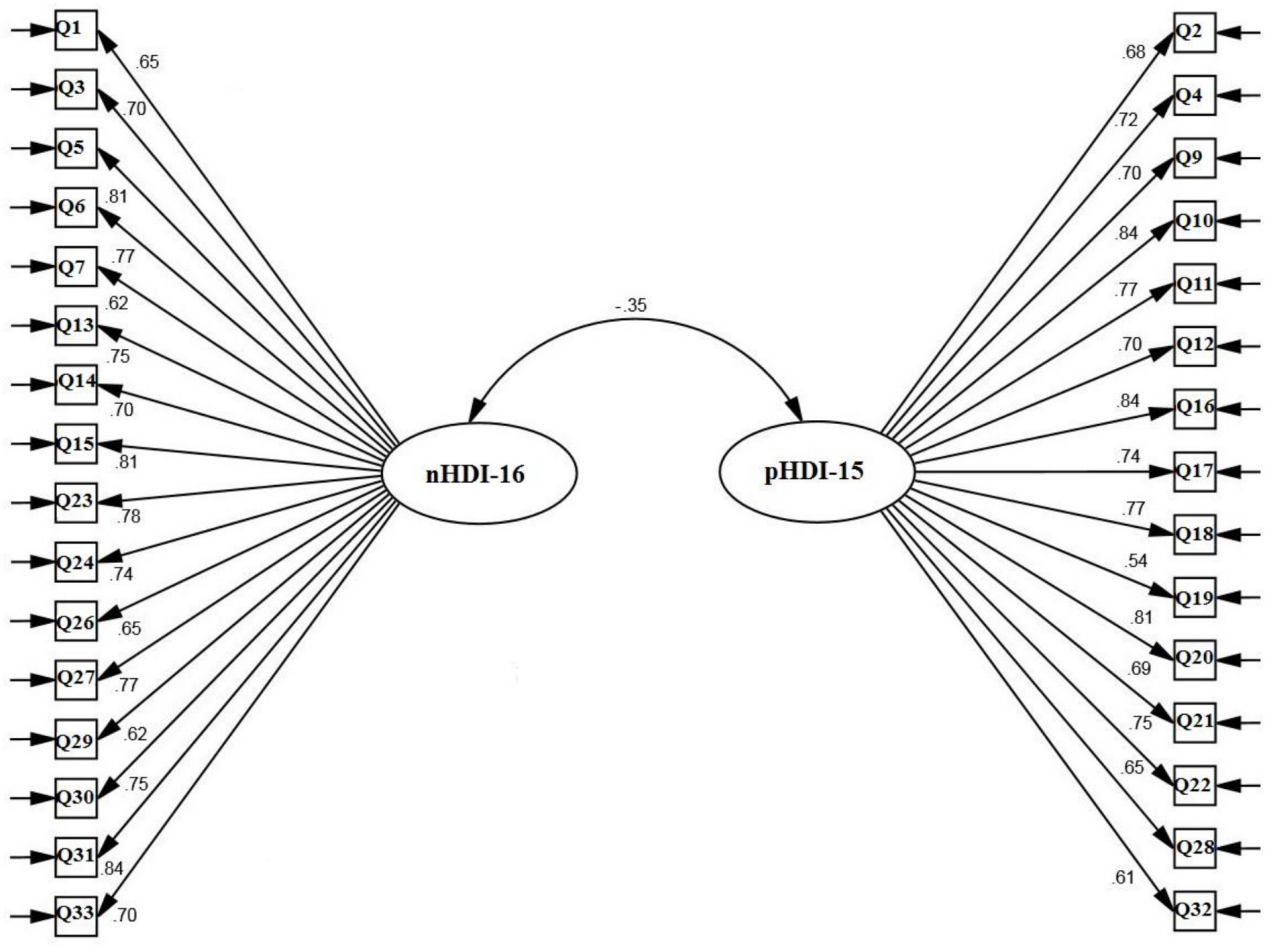
Confirmatory factor analysis of food frequency consumption scale.

The fit indices demonstrated that the two-factor construct of FFC had a good and acceptable fit in the Iranian adult community (S-B χ2 = 1,099.864, DF = 428, P < 0.001, CFI = 0.963, TLI = 0.948, RMSEA = 0.047 (90% CI: 0.042-0.051), SRMR = 0.031). All factor loadings of items on the FFC scale were significant on their factors (all ps < 0.001) (Table 2, Figure 1).

### Reliability (internal consistency and stability) and convergent validity

The internal consistency (Cronbach's alpha, McDonald's Ω, and θ), CR and ICC values of the two FFC subscales (pHDI and nHDI) are listed in Table 3. The AVE values of the two FFC subscales were >0.5 and < CR>0.7 (Table 2). There was a moderate and negative correlation between the two FFC subscales as well as there was a moderate to a high correlation between the pHDI and nHDI subscales with the NB scale (Table 3).

**Table 3:** Correlation and reliability (internal consistency and test-retest) of two scales of food frequency consumption and nutrition beliefs.

**Variable**	**α**	**Θ**	**Ω**	**ICC(95% CI)**	***P*-value**	**1**	**2**
**pHDI-15**	0.939	0.936	0.932	0.928 (0.918–0.935)	< 0.001	1	
**nHDI-16**	0.896	0.894	0.890	0.846 (0.831–0.863)	< 0.001	−0.302	1
**Nutrition beliefs**	0.935	0.933	0.930	0.917 (0.901–0.947)	< 0.001	0.556	−0.447

α, Cronbach's alpha coefficients; θ, theta coefficient; Ω, McDonald omega coefficient; ICC, intra-class correlation.

The results demonstrated that there was a significant difference between the 4 dietary indices (hSFDI-8, hSDI-4 LGIDI-4 and hGIDI-7) in terms of BMI and education. Scores on these indices were higher in those with bachelor's and higher degrees than in those with less than a bachelor's degree, and scores on the hSFDI-8, hSDI-4, and hGIDI-7 indices were higher in obese adults than in others. The hGIDI-7 index was higher for men than for women, higher for divorced and widowed persons than for married and single persons, and higher for adults with insufficient income than for persons with other income levels. The LGIDI-4 index was higher for women than for men and higher for persons with sufficient or higher income than for persons with other income levels. The hSDI-4 index was higher for men than for women, higher for divorced and widowed adults than for married and single adults, higher for persons with insufficient income than for persons with other income levels, and higher for adults aged 30–40 than for other age groups. The hSFDI-8 index was higher for divorced and widowed individuals than for married and single persons, and for adults with sufficient income than for individuals with other income levels. Based on the results, the dietary indices differed significantly in the levels of the demographic variables. Therefore, these indices together with two pHDI and nHDI indices could be considered as dietary indices (Table 4).

**Table 4:** Changes in dietary indexes (hSFDI-8, hSDI-4, LGIDI-4, hGIDI-7), at the levels of demographic variables.

**Variable**		**Dietary indexes**			
		**hgidi-7 (%)**	**LGIDI-4 (%)**	**hSDI-4 (%)**	**hSFDI-8 (%)**
BMI^a^	Normal weight	18.72 (0.71)	19.22 (0.82)	16.54 (0.69)	9.05 (0.4)
	Overweight	17.49 (0.6)	17.92 (0.63)	16.39 (0.65)	12.10 (0.45)
	Obese	20.10 (0.78)	15.14 (0.56)	20.96 (0.92)	15.73 (0.69)
*P* value		0.043	< 0.001	< 0.001	< 0.001
Gender^b^	Male	20.37 (0.61)	16.19 (0.47)	18.93 (0.62)	11.39 (0.39)
	Female	16.92 (0.52)	18.13 (0.58)	16.30 (0.58)	12.26 (0.43)
*P* value		< 0.001	0.011	0.002	0.143
Marital status^a^	Married	17.82 (0.53)	16.86 (0.48)	16.95 (0.57)	11.12 (0.38)
	Single	17.89 (78)	16.70 (0.74)	16.09 (0.79)	11.13 (0.53)
	Widowed	21.28 (1.16)	18.31 (1.25)	21.52 (1.35)	15.86 (1.12)
	Divorced	24.30 (1.56)	20.37 (1.56)	23.80 (1.53)	15.75 (1.07)
*P* value		< 0.001	0.079	< 0.001	< 0.001
Education level^a^	Diploma and under diploma	16.73 (0.56)	14.55 (0.47)	15.54 (0.6)	11.03 (0.44)
	Associate Degree	19.25 (1.02)	16.21 (0.91)	18.39 (1.07)	13.04 (0.75)
	Bachelor	19.16 (0.75)	19.48 (0.74)	18.12 (0.78)	11.77 (0.52)
	Master of arts	22.72 (1.34)	22.07 (1.38)	22.16 (1.43)	13.22 (0.89)
*P* value		< 0.001	< 0.001	< 0.001	0.033
Income status^a^	Insufficient	22.54 (0.93)	15.33 (0.63)	20.90 (0.91)	10.23 (0.48)
	Sufficient	17.35 (0.45)	17.89 (0.48)	16.39 (0.5)	12.52 (0.38)
	>sufficient	15.62 (1.20)	17.51 (1.52)	16.33 (1.41)	11.42 (0.9)
*P* value		< 0.001	0.013	< 0.001	0.003
Year (age)^a^	< 30	18.77 (0.75)	17.56 (0.65)	17.53 (0.79)	12.03 (0.49)
	30–40	19.11 (0.55)	16.95 (0.53)	18.73 (0.58)	12.01 (0.41)
	>50	16.53 (0.91)	17.20 (0.94)	13.84 (0.95)	10.92 (0.77)
*P* value		0.082	0.773	< 0.001	0.404

High-Glycemic-Diet-Index-7 (hGIDI-7); Low-Glycemic-Diet-Index-4 (LGIDI-4); High-Sugar Diet-Index-4 (hSDI-4); High-Saturated-Fats-Diet-Index-8 (hSFDI-8). ^a^Analysis of Variance (ANOVA). ^b^Independent Samples T Test.

### Nutrition beliefs (NB)

#### Construct validity

##### EFA

The results of evaluating sampling adequacy indices (KMO) and Bartlett's sphericity test were 0.955 and 9,974.22 (*P* < 0.001), respectively. One factor with a eigenvalue values >1 was identified for the NB scale and confirmed based on the screen plot diagram. The data were rotated by Varimax rotation, and in total, one factor of NB was 46.61% of the total variance.

The findings suggested that factor loadings of all items (except items 55 and 56) were >0.3; hence, two items “once-daily consumption of cereals is sufficient (Q1)” and “Only children and adolescents should drink milk (Q2)” were deleted. The correlation between all items and the total score was >0.3 (Table 5).

**Table 5:** Results of the exploratory and confirmatory factor analysis of the nutrition beliefs scale.

**No**	**Items**	**Internal consistency** **(α: ITC)**	**EFA sample 1** ***n*** = **700**			**CFA sample 2** ***n*** = **700**	
			**Factor loading**	**h^2^**	**Eigenvalue (%variance explained)**	**λ_X_**	**(AVE; CR)**
3	once-daily consumption of cereals is sufficient	(0.930; 0.754)	0.795	0.632	10.722 (46.615%)	0.77	0.512 (0.959)
4	Eating moldy bread can lead to food poisoning caused by Salmonella.	(0.931; 0.719)	0.763	0.583		0.75	
5	High salt intake prevents highpertention.	(0.932; 0.666)	0.721	0.519		0.71	
6	Limiting the intake of high-fat foods prevents cardiovascular disease.	(0.931; 0.734)	0.778	0.605		0.76	
7	Frequent consumption of fatty fish (such as salmon) can lead to clogged arteries.	(0.931; 0.731)	0.781	0.611		0.78	
8	Frequent consumption of grilled meats can lead to cancer.	(0.930; 0.754)	0.793	0.629		0.78	
9	A vegetarian diet increases the risk of anemia.	(0.931; 0.693)	0.751	0.564		0.75	
10	Natural yogurts contain beneficial intestinal bacteria.	(0.930; 0.736)	0.780	0.608		0.78	
11	Vegetable and olive oils are high in cholesterol.	(0.931; 0.669)	0.723	0.523		0.74	
12	Whole-wheat bread has more fiber than white bread.	(0.935; 0.437)	0.457	0.209		0.51	
13	Fruits and vegetables are calorie free.	(0.931; 0.704)	0.756	0.571		0.76	
14	Enriched animal and vegetable butters contain high amounts of vitamins A and D.	(0.935; 0.398)	0.404	0.163		0.56	
15	Low-fat cheeses have less calcium than regular cheeses.	(0.931; 0.735)	0.782	0.612		0.78	
16	Kalle pache (Khash) has high levels of cholesterol (harmful fats).	(0.935; 0.447)	0.462	0.213		0.51	
17	In a healthy diet, complex carbohydrates such as whole cereals (rice and pasta) should be replaced with simple sugars (sugar, cakes and pastries).	(0.931; 0.707)	0.759	0.576		0.75	
18	In a balanced diet, the main source of energy should be provided through protein intake.	(0.930; 0.781)	0.815	0.663		0.79	
19	Inadequate intake of niacin leads to skin inflammation and diarrhea.	(0.930; 0.768)	0.808	0.653		0.80	
20	sunlight exposure increases the synthesis of vitamin D in the body.	(0.935; 0.426)	0.440	0.194		0.59	
21	Phosphorus is one of the main components of neural tissue.	(0.930; 0.756)	0.797	0.635		0.78	
22	In a healthy diet, the ratio of calcium to phosphorus should be equal.	(0.936; 0.368)	0.382	0.146		0.54	
23	Consumption of fruits containing high amounts of vitamin C leads to increased absorption of iron in the body.	(0.935; 0.401)	0.419	0.176		0.57	
24	Cooking vegetables in cold water helps preserve the nutrients.	(0.936; 0.387)	0.390	0.152		0.74	
25	Sweets and animal fats are rich in nutrients.	(0.932; 0.651)	0.696	0.484		0.68	

EFA, Exploratory Factor Analysis; CFA, Confirmatory Factor Analysis; Internal Consistency (Cronbach's Alpha if item Deleted and Item-Total Correlation [ITC]); λx, standardized coefficients; h2, Communalities.

##### CFA

**Figure 2:**
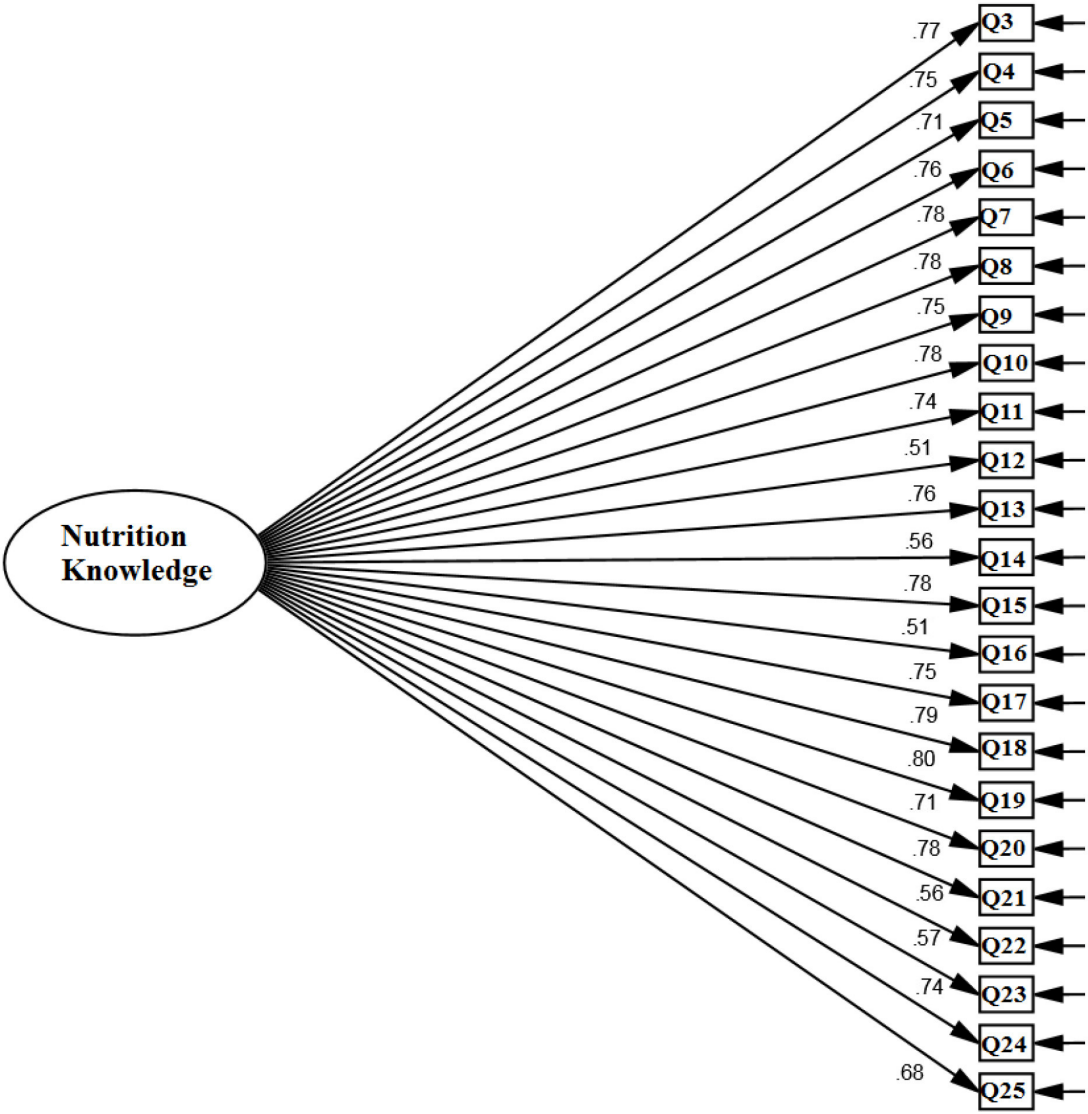
Confirmatory factor analysis of the nutrition beliefs scale.

The fit indices displayed that the one-factor construct of NB had a good and acceptable fit in the Iranian adult community (S-B χ^2^ = 446.304, DF = 217, *P* < 0.001, CFI = 0.923, TLI = 0.920, RMSEA = 0.039 (90% C.I: 0.03-0.05), SRMR = 0.031). All factor loadings of items on the NB scale were significant (all ps < 0.001) (Table 5; Figure 2).

### Reliability (internal consistency and stability) and convergent validity

The internal consistency (α, Ω, and θ) and CR values of the NB scale were >0.7. For the NB scale, the ICC was 0.917 (Table 3). The AVE values for the NB scale were >0.5 and CR>0.7 (Table 5).

#### Life style

The lifestyle scale had 18 items, of which 16 items were analyzed, and two items “Please provide the type of diet and” How long have you been following this diet?” were not analyzed because each participant could choose more than one option. According to test-retest results, the percentage of participants classified at the item level was on average 93.96%. Among the studied adults, the highest cross-classification agreement was 99.3 and 89.3% for the items “How would you describe your knowledge of nutrition?” and "Are you currently on a special diet (to lose or gain weight)?,” respectively. Cross-classification analysis demonstrated that on the DH scale, the percentage of correct classification was high, and the percentage of misclassification was very low among the studied persons. Kappa value ranged from 0.772 (for item “Are you currently using industrial addictive substances such as hashish, marijuana, drug flowers, crack and methamphetamine?” to 0.989 (for item “How would you describe your knowledge of nutrition?”), respectively. The Kappa statistic was >0.4 for all analyzed items and its value was acceptable (Table 6).

**Table 6:** Agreement and misclassification in test-retest for lifestyle scale.

**No**.	**Questionnaire items**	**Cat**.	**Total agreement**	**Misclassification**			**Kappa**
				**1 ±cat**.	**2 ±cat**.	**3 ±cat**.	
1	Are you currently on a special diet (to lose or gain weight)?	3	89.3	10	0.7		0.481
4	How often do you eat out, for example in a restaurant, cafe or canteen?	7	95.3	1.4	2	1.3	0.929
5	Do you currently drink alcohol?	2	96.6	2.7	0.7		0.765
6	Do you currently smoke cigarettes, pipe or hookah?	2	94.6	5.4			0.706
7	Did you use to smoke cigarettes, pipe or hookah	2	98.6	1.4			0.882
8	Are you currently using drugs such as poppy, opium, opium juice (Shireh), methadone, bupropion and heroin?	2	95.3	4.7			0.813
9	Are you currently using industrial addictive substances such as hashish, marijuana, drug flowers, crack and methamphetamine?	2	94	6			0.772
10	How many hours do you sleep on weekdays?	3	97.3	2.7			0.953
11	How many hours do you sleep on the weekends?	3	96.6	3.4			0.947
12	How many hours a day do you spend watching TV/using a computer or mobile phone for entertainment or work?	6	98	2			0.969
13	How would you describe your physical activity at work/school or university?	3	97.3		0.7	2	0.955
14	How would you describe your physical activity in your spare time?	3	97.3	2.7			0.955
15	How would you describe your health compared to your peers?	3	94.6	2	3.4		0.904
16	How would you describe your knowledge of nutrition?	4	99.3			0.7	0.989
17	How would you describe your diet?	4	96.6	1.3	1.3	0.8	0.938
18	How would you describe your diet on weekdays compared to weekends?	3	93.3	0.7	4	2	0.895

No., item number in the questionnaire. Cat., number of response categories in the question. evaluated in 3 categories: low (0–33 points), moderate (34–66 points), high (67–100 points).

## Discussion

The aims of the current study were to translate the KomPAN questionnaire and evaluate its psychometric properties in Iranian adults and measure DQS and 4 indicators including hGIDI-7, LGIDI-4, hSDI-4 and hSFDI-8 based on 3 groups of body mass index (BMI) (BMI = 18.5–24.9, BMI = 25–29.9 and BMI ≥ 30), gender, educational level, income status, and age.

Because of cultural and social differences between our samples and the ones form Kowalkowska's study (12) some changes were made to the items. Some of the food categories in the original set was accessible to Iranian adults, hence the categories that were more accessible and commonly used among Iranians were substituded. This made the tool to become a more general and practical too especially among Iranian and Spanish communities. In this study, some of the items were eliminated during the psychometric process and some items were added to the tool. Before the psychometric process and after the translation. This has been added to the discussion section.

### DH

The results of the cross-classification analysis showed that most of the items on the DH scale were correctly classified in two repetitions and only 6.04% of the items were misclassified, indicating that the items were capable of measuring the DH construct. Among the studied adults, the highest cross-classification agreement was related to the items “What type of milk (pasteurized high- or low-fat milk or whole milk) and dairy products do you usually consume?” and “Do you add sweeteners to hot drinks like tea, hot chocolate and coffee?,” respectively, representing that these two items were more important than other items in measuring DH. Kappa values for all items analyzed were >0.4, illustrating the acceptable reliability of this scale (29), which is consistent with the results of the study of Kowalkowska et al. (12).

### FFC and NB

The results of KMO and Bartlett's sphericity test suggested that the data for FFC and NB scales were appropriate for EFA. Klein (32) found that EFA and CFA require 10 samples per item and a minimum of 200 samples, respectively. The fit indices showed that the two-factor construct of the FFC scale and one-factor construct of the NB scale had a good and acceptable fit in the Iranian adult community. On the FFC scale except for two items “How often do you use lard to flavor your bread or for cooking? ” and “How often do you eat tinned meats?” as well as on the NB scale, except for two items “once-daily consumption of cereals is sufficient.” and “Only children and adolescents should drink milk.,” the correlation between all items and the total score was higher than the minimum acceptable value of >0.3 (27). The internal consistency and CR values of the two FFC subscales (pHDI and nHDI) and NB scale were higher than the recommended value of 0.7 (33), indicating good reliability of these two scales. The ICC values for the FFC subscales and NB scale were higher than the recommended value of 0.8 (31), representing the repeatability of these two scales. AVE and CR values of two FFC subscales and NB scale were >0.5 and >0.7, respectively. In addition, for both scales, CR values were >AVE, and according to the Fornell-Larcker criterion (1981) criteria, these two scales had good convergent validity. The results of the ongoing study revealed that there was a moderate and negative correlation between the two subscales of pHDI and nHDI, displaying that the FFC scale consisted of two independent constructs. Moreover, a significant positive and negative correlation was found between pHDI and nHDI with the NB scale, respectively, indicating that both FFC and NB scales had good discriminant validity (27).

### Lifestyle

The results of the cross-classification analysis showed that most items of the lifestyle scale were correctly classified in the primary class in two repetitions and only 4.13% of items were incorrectly classified in another class in the second time. This shows that the items of lifestyle structure have an acceptable reproducibility. Among the studied adults, the highest cross-classification agreement was related to the items “How would you describe your knowledge of nutrition?” and "Are you currently on a special diet (to lose or gain weight)?,” indicating that these two items are more important than other items in measuring lifestyle. Kappa values for all analyzed items were >0.4, representing the acceptable reliability of this scale (29). which is consistent with the results of the study of Kowalkowska et al. (12).

## Conclusion

It is recommended that other researchers use the KomPAN questionnaire due to its simplicity, comprehensibility, multidimensionality and acceptable validity and reliability to identify DH, FFC, NB and lifestyle as well as measure DQS in the adult community. Moreover, it is proposed to use hGIDI-7, LGIDI-4, hSDI-4 and hSFDI-8 indices as dietary indices in addition to pHDI and nHDI indices.

### Strengths of the study

The translation and evaluation of the psychometric properties of the KomPAN questionnaire in the current study enable other researchers to utilize this tool to identify dietary patterns in adults. DQS can be measured in the target population using the FFC scale of this questionnaire. A large sample size for EFA and CFA is another strength of the present study. The new classification of the items of the FFC scale based on the food culture and lifestyle of the Iranian community in this study will help researchers to measure FFC more accurately and comprehensively. In the present study, other dietary indices including hGIDI-7, LGIDI-4, hSDI-4 and hSFDI-8 were evaluated and had a good validity based on the analysis of known groups (BMI, gender, educational level, income status, and age). Using a weight estimator for CFA is another strength of the current study.

### Limitations

The self-report version of the KomPAN questionnaire was applied to assess dietary habits, FFC, NB and lifestyle in healthy individuals. Therefore, it is proposed to use two IA-Q and self- SA-Q versions of the KomPAN questionnaire to increase the validity of the data. It is also recommended that the psychometric properties of the KomPAN questionnaire should be evaluated in other people with physical problems. The use of a convenient sampling method may limit the generalizability of the results to adults living in other regions of Iran. It is recommended to evaluate the psychometric properties of the KomPAN questionnaire in a more heterogeneous population, in different regions of Iran with various cultures and different social and demographic characteristics. In the present study, the measurement invariance, concurrent validity and common factor bias of the KomPAN questionnaire were not investigated. Hence, it is recommended that these indices be evaluated to increase the validity of the tool in future studies in different populations. Another limitation of the ongoing study is that the number of items in the KomPAN questionnaire is very large, which may cause respondents to become tired while completing the questionnaire, especially in the last parts of the instrument. Therefore, it is recommended to change the order of the sections of the KomPAN questionnaire during data collection.

## Data availability statement

The original contributions presented in the study are included in the article/supplementary material, further inquiries can be directed to the corresponding author.

## Ethics statement

The current study was approved by the Ethics Committee of Babol University of Medical Sciences (IR.MUBABOL.HRI.REC.1400.046). The patients/participants provided their written informed consent to participate in this study.

## Author contributions

KS and FG conceptualized the research project. FG, KJ, FC, MK, and KS performed data curation, analysis, interpreted analysis results, and wrote first draft of the manuscript. FC, KJ, AS, RG, MK, and ZP reviewed and edited the manuscript. All authors read, revised, and approved the final manuscript.

## Funding

This study was funded by the Babol University of Medical Sciences. The funder had no role in study design, data collection, data analysis and decision to publish or preparation of the manuscript.

## Conflict of interest

The authors declare that the research was conducted in the absence of any commercial or financial relationships that could be construed as a potential conflict of interest.

## Publisher's note

All claims expressed in this article are solely those of the authors and do not necessarily represent those of their affiliated organizations, or those of the publisher, the editors and the reviewers. Any product that may be evaluated in this article, or claim that may be made by its manufacturer, is not guaranteed or endorsed by the publisher.

## Abbreviations

hSFDI-8: high-saturated-fats-Diet-Index-8
hSDI-4: high-Sugar-Diet-Index-4
LGIDI-4: low-Glycaemic-Diet-Index-4
hGIDI-7: high-Glycaemic-Diet-Index-7
BMI: Body mass index
NB: Nutrition beliefs
FFC: Food frequency consumption
DH: Dietary habits
EFA: Exploratory factor analysis
CFA: Confirmatory factor analysis
ICC: Intraclass correlation coefficient
IA-Q: Interviewer-administered
SA-Q: Self-administered
DQS: Diet quality scores
pHDI: pro-Healthy-Diet-Index
nHDI: non-Healthy-Diet-Index
CVR: Content validity ratio
CVI: Content validity index
KMO: Kaiser-Meyer-Olkin
PAF: Principal axis factoring
GFI: Goodness of fit indices
CFI: Comparative fit index
TLI: Tucker–Lewis index
SRMR: Standardized root mean square residual
RMSEA: Root mean square error of approximation
CI: Confidence interval.
